# Image Preprocessing with Enhanced Feature Matching for Map Merging in the Presence of Sensing Error

**DOI:** 10.3390/s23167303

**Published:** 2023-08-21

**Authors:** Yu-Lin Chen, Kuei-Yuan Chan

**Affiliations:** Department of Mechanical Engineering, National Taiwan University, Taipei 106319, Taiwan; chenyl@solab.me.ntu.edu.tw

**Keywords:** simultaneous localization and mapping, map merge, image stitching, occupancy grid map

## Abstract

Autonomous robots heavily rely on simultaneous localization and mapping (SLAM) techniques and sensor data to create accurate maps of their surroundings. When multiple robots are employed to expedite exploration, the resulting maps often have varying coordinates and scales. To achieve a comprehensive global view, the utilization of map merging techniques becomes necessary. Previous studies have typically depended on extracting image features from maps to establish connections. However, it is important to note that maps of the same location can exhibit inconsistencies due to sensing errors. Additionally, robot-generated maps are commonly represented in an occupancy grid format, which limits the availability of features for extraction and matching. Therefore, feature extraction and matching play crucial roles in map merging, particularly when dealing with uncertain sensing data. In this study, we introduce a novel method that addresses image noise resulting from sensing errors and applies additional corrections before performing feature extraction. This approach allows for the collection of features from corresponding locations in different maps, facilitating the establishment of connections between different coordinate systems and enabling effective map merging. Evaluation results demonstrate the significant reduction of sensing errors during the image stitching process, thanks to the proposed image pre-processing technique.

## 1. Introduction

Autonomous exploration involves the process of robots constructing maps of unfamiliar environments by sensing their surroundings while navigating. Simultaneous Localization and Mapping (SLAM) is a widely adopted approach in autonomous exploration as it enables robots to simultaneously build maps of the environment and determine their own location. The concept of SLAM was initially proposed and discussed in 1986, and it has since gained widespread recognition as a valuable technique [1,2]. Over the years, several commonly used methods have been proposed, starting from the 1990s until the present day. These include EKF-SLAM [3], Fast-SLAM [4], GraphSLAM [5], and others, which have all contributed significantly to the field of SLAM.

Despite advancements in SLAM technology, a single robot is not sufficient to conduct a comprehensive environmental exploration of a large area. As a result, researchers have focused on the development of multi-robot systems (MRS) for the decentralized exploration of environments. MRS involve collaboration among distributed robots, which enhances both work efficiency and system robustness when compared to single-robot systems. However, local maps generated by multiple robots may have different coordinates, making it challenging to integrate them into a global map. Therefore, researchers have explored methods for combining various local maps, which is known as the map merging problem.

This study presents a map merging method that utilizes image features to address the issue of sensing errors, which has received little attention in the literature. Our approach involves using image processing techniques to correct local maps before merging them into a global map. This paper is organized as follows: Section 2 reviews previous methods proposed to address the map merging problem. Section 3 presents a detailed problem statement. In Section 4, we describe our proposed method. Section 5 presents the simulation results and provides a discussion. Finally, Section 6 concludes the study and offers suggestions for future work.

## 2. Literature Review

Robot mapping utilizes different types of maps, and the methods for merging them may vary. The commonly used maps include occupancy grid maps, feature maps, and topological maps [6]. Occupancy grid maps are widely used and can be easily implemented in any environment [7]. Feature maps require less memory space, but their complex feature extraction process consumes computational resources. Topological maps depict a network structure and are useful for specific tasks such as navigation and path planning. In this study, we use occupancy grid maps as they have abundant applications in the literature.

Map merging methods can be classified as direct or indirect, with most recent methods using indirect approaches. Lee’s survey reviewed several merging methods and classified them into these two types based on their logical structure [8]. Direct methods involve establishing correlations between the sensing data of multiple robots to obtain their relative positions for merging. They can be based on direct observation, where robots use their sensors to observe cooperating robots and determine their relative positions [9], or on identifying common environmental objects, such as ceiling features, to establish relative positions [10]. However, direct methods have the limitation of requiring robots to be in the same field, which restricts their activity range and time allocation.

Indirect methods offer a solution to the limitations of direct methods but come at the expense of increased computational complexity. These methods treat maps as images or 2D matrices, allowing them to overcome temporal and spatial constraints and reduce computational requirements by discarding certain sensing data [8]. Indirect methods often extract and match features to establish correlations between the maps. Once these correlations are obtained, a relative transformation can be calculated by scaling, rotating, and translating the grid cells of the maps. Indirect methods effectively align maps with significant deviations in position or orientation, enhancing their reliability for map merging purposes.

The map images exhibit a diverse range of features, encompassing spectral features of line segments [11] and local extreme features [12]. Depending on the extracted features from these maps, different methods are employed. Carpin [11] proposed a non-iterative and fast algorithm that utilizes the Hough transform to determine the necessary rotation and translation parameters for map merging. This approach performs well in spatially structured environments, such as square rooms. However, it is important to note that this method has certain limitations, such as the requirement for local maps to have the same scale and a sufficient number of overlapping areas for successful merging. On the other hand, Ferrão [12] utilized the scale invariant feature transformation (SIFT) algorithm [13] to extract robust features from occupancy grid maps. This widely-used algorithm identifies extreme grid points in map images and performs feature matching. Although SIFT is commonly employed in computer vision and image stitching problems [14], it is worth noting that typical red-green-blue (RGB) images exhibit a higher diversity of texture, brightness, and color characteristics, enabling the extraction of more features compared to occupancy grid maps with pixel values represented by only three integers denoting free, unknown, and occupied areas. Furthermore, the presence of sensor errors and uncertainties in occupancy grid maps poses additional challenges for feature extraction and matching, leading to a decreased success rate of map merging solutions, as discussed in Jiang’s works [7,15].

In order to overcome the limitations in feature extraction and matching from occupancy grid maps, we present a novel image pre-processing operation aimed at reducing the impact of sensor errors and uncertainty. Our objective is to improve the effectiveness of feature extraction and ultimately address the challenge of map merging. Our proposed map merging approach involves two key steps. Firstly, we apply the image pre-processing technique to mitigate the impact of uncertainty and enhance the efficiency of feature extraction. This step aims to extract significant features from the occupancy grid maps. Secondly, we utilize an image stitching method to merge the pre-processed occupancy grid maps. By combining these steps, we aim to achieve a more effective and reliable map merging solution.

## 3. Basic Definitions on Map Merging

**Figure 1:**
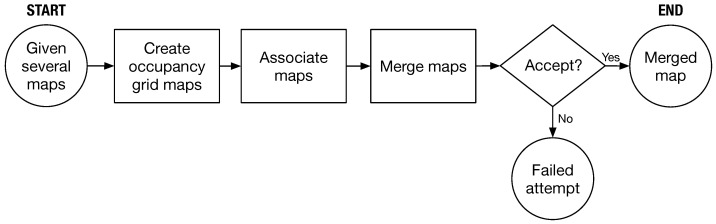
Basic concept of map merging.

Consider multiple map images obtained by robots. Figure 1 illustrates the process defining the map merging problem in this work. After the maps are obtained, occupancy grid maps are generated based on the availability of each grid element. Two maps are associated by transforming them onto the same basis. The association results in a merging index, which determines whether the maps can be merged into one. If the index is acceptable, the maps are merged, and if not, the merging attempt fails. The rest of this section describes the occupancy grid map, the transformation between two maps, and the index used to evaluate the merged result in detail.

**Figure 2:**
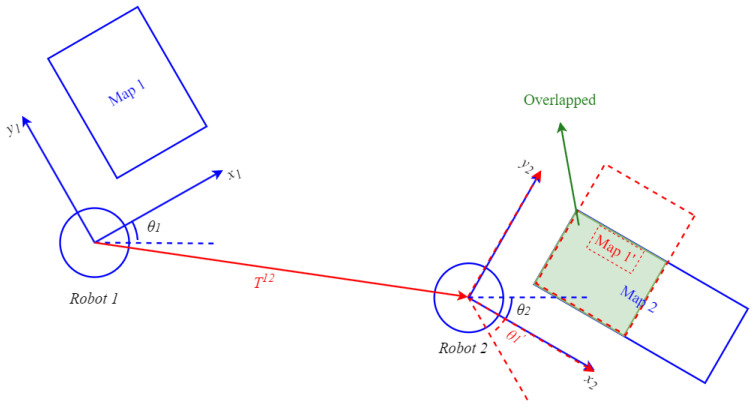
Diagram of overlapping regions of maps $M_{1}$ and $M_{2}$. Original coordinates are expressed by blue solid lines, and transformed coordinates are expressed by red dashed lines.

- Create occupancy grid maps: In the context of map merging, we define *M* as a 2D occupancy grid map with size r×c as shown in Equation (1). Each cell $m_{ij}$ contains a binary value that indicates whether the cell is occupied ($m_{ij}=1$) or free ($m_{ij}=0$).
  (1) $$M=\left[\begin{array}{ccc} m_{11} & \cdots & m_{1c}\\ \vdots & \ddots & \vdots \\ m_{r1} & \cdots & m_{rc}. \end{array}\right]$$
  Let $p(m_{ij}=1)$ be the probability that the cell is occupied. A grid cell could be classified into three conditions:
  $$m_{ij}=\left\{\begin{array}{cc} \mathrm{occupied},\; & \mathrm{if}\;p\left(m_{ij}=1\right)\geq p_{\mathrm{occupied}}.\\ \mathrm{free},\; & \mathrm{if}\;p\left(m_{ij}=1\right)\leq p_{\mathrm{free}}.\\ \mathrm{unknown},\; & \mathrm{else}, \end{array}\right.$$
  where $p_{\mathrm{occupied}}$ and $p_{\mathrm{free}}$ are two predefined thresholds.
  In addition to a binary value, if *M* represents an image, each cell element in Equation (1) will have a pixel value that can also be used to represent the cell conditions.
  $$m_{ij}=\left\{\begin{array}{cc} 0\; & \mathrm{if}\;\mathrm{cell}\;\mathrm{type}\;\mathrm{is}\;“\mathrm{occupied}.”\\ 255\; & \mathrm{if}\;\mathrm{cell}\;\mathrm{type}\;\mathrm{is}\;“\mathrm{free}.”\\ 128\; & \mathrm{if}\;\mathrm{cell}\;\mathrm{type}\;\mathrm{is}\;“\mathrm{unknown}.” \end{array}\right.$$
- Associate maps for merging: Consider two maps $M_{1}$ and $M_{2}$ with different coordinates, as shown in Figure 2. The transformation matrix between them is denoted as $T^{12}\left(s,\theta ,t_{x},t_{y}\right)$, as defined in Equation (2).
  (2) $$T^{12}\left(s,\theta ,t_{x},t_{y}\right)=\left[\begin{array}{ccc} s\cdot \cos\theta & -s\cdot \sin\theta & t_{x}\\ s\cdot \sin\theta & s\cdot \cos\theta & t_{y}\\ 0 & 0 & 1 \end{array}.\right]$$
  In Equation (2), *s* represents the scale difference between the coordinates of the maps, θ represents the angle difference, and $t_{x}$ and $t_{y}$ represent the translation difference. Using this transformation, map $M_{1}$ can be transformed into $M_{1}^{\prime }$ and overlapped with parts of map $M_{2}$, as illustrated in Figure 2.
- Determine acceptance of the merged result: In Equation (3), the acceptance index [11], denoted as $\omega (M_{1},M_{2})$, serves as a means to evaluate the merging result when the transformed map $M_{1}^{\prime }$ is overlaid onto map $M_{2}$. The acceptance index considers two types of grids: *agreement* and *disagreement*. *Agreement* represents correctly paired grid cells between $M_{1}$ and $M_{2}$, indicating that both maps have the same type (occupied-occupied or free-free) in the overlapping areas. On the other hand, *disagreement* signifies incorrect pairings, indicating that the two maps have different types (occupied-free) in those regions. Due to its higher uncertainty, the unknown type is not discussed here.
  When the transformation $T^{12}$ accurately aligns the grids of the transferred map $M_{1}^{\prime }$ with those of map $M_{2}$ in the overlapping regions, the number of *agreement* grids increases while the number of *disagreement* grids decreases. As a result, the acceptance index $\omega (M_{1}^{\prime },M_{2})$ approaches 1. Conversely, if the transformation $T^{12}$ is incorrect, the overlapping areas will contain numerous disagree grids, leading to a smaller or even 0 acceptance index $\omega (M_{1}^{\prime },M_{2})$. Thus, the acceptance index effectively determines the correctness of the map transformation and reflects the quality of the merging result. In our study, we define an index threshold, as shown in Equation (4), to judge the accuracy of the map transformation.
  (3) $$\begin{array}{cc} \omega & \left(M_{1},M_{2}\right)=\left\{\begin{array}{ccc} 0, & \; & \mathrm{if}\;agr(M_{1},M_{2})=0\\ \frac{agr(M_{1},M_{2})}{agr\left(M_{1},M_{2}\right)+dis\left(M_{1},M_{2}\right)}, & \; & \mathrm{if}\;agr(M_{1},M_{2})\neq 0 \end{array}\right. \end{array}$$
  (4) $$\omega \left(M_{1}^{\prime },M_{2}\right)\geq \omega_{\mathrm{thres}}.$$

With an acceptable merged result, we can successfully combine multiple images from robots into a single map. In this study, we use a threshold of $\omega_{\mathrm{thres}}=0.95$ and test it in various scenarios. In the next section, we will present our method that builds on the concept flow shown in Figure 1 to handle images that may contain sensing errors in reality.

## 4. Map Merging Method

In this section, we present our novel image pre-processing method, which effectively mitigates the impact of sensing errors and uncertainties in maps prior to their stitching. The proposed pre-processing operation focuses on eliminating noise and distortion issues in the occupancy grid map through several image processing steps. By applying these steps, we ensure that the existing image-stitching method is unaffected by noise and can seamlessly merge the maps. In what follows, we will describe the existing process of imaging stitching in Section 4.1, and our proposed operation in Section 4.2.

### 4.1. Existing Method in Image Stitching

**Figure 3:**
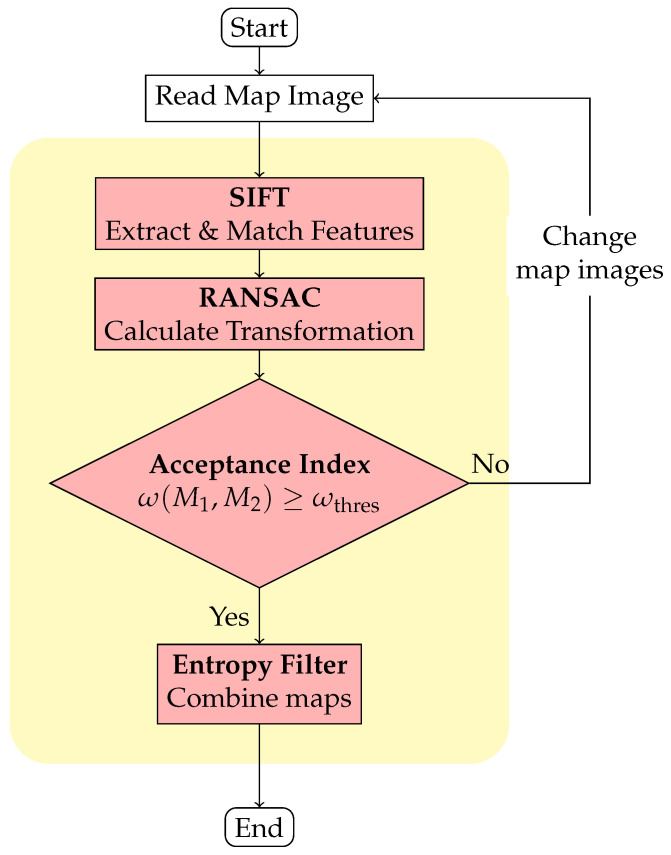
Flowchart of the image stitching technique.

We utilize an image-stitching method [16] proposed by Mills for our stitching operation. However, when combining two occupancy grid maps, it is essential to consider their probabilistic information, which differs from regular images. Therefore, we incorporate an entropy filter [17] after calculating the relative transformation using Mills’ method. The complete operation is illustrated in Figure 3. In this operation, two maps $M_{1}$ and $M_{2}$ are merged, as shown in Figure 2. First, the scale-invariant feature transform (SIFT) [18,19] is used to extract features from the occupancy grid maps. SIFT applies Gaussian filters to build the scale space of the image and then finds the extreme points that can be used as features under the difference of Gaussians (DoG) in the scale space. The gradient vector around each feature is calculated, and a descriptor with the histogram of the gradient vector is obtained. Therefore, SIFT features are invariant to scale, rotation, and brightness [18,19]. The SIFT algorithm obtains features $P=\{p_{1},\ldots ,p_{n}\}$ from $M_{1}$ and features $Q=\{q_{1},\ldots ,q_{m}\}$ from $M_{2}$. These features are matched as pairs $\left\{p_{i}=\left(x_{pi},y_{pi}\right),q_{j}=\left(x_{qj},y_{qj}\right)\right\}$, i=1,…,n and j=1,…,m by using the Euclidean distance to find the feature $q_{j}$ in *Q* that is closest to the feature $p_{i}$ in *P*.

Matching pairs are further processed by applying the random sample consensus algorithm (RANSAC) [20] to eliminate outliers and estimate a relative transformation between the maps. RANSAC effectively exclude outliers and calculate a model that fits the input data by randomly sampling the data. If the inliers (that conform to the real data model) are selected, the correct model can be calculated and outliers can be effectively excluded. Specifically, in our method, RANSAC includes the following three steps.

Step 1 Define a ‘Guess’ model.
  (5) $$\begin{array}{cc} T^{12,\mathrm{guess}} & \left(s,\theta ,t_{x},t_{y}\right)=\left[\begin{array}{ccc} s\cos\theta & -s\sin\theta & t_{x}\\ s\sin\theta & s\cos\theta & t_{y}\\ 0 & 0 & 1. \end{array}\right] \end{array}$$
  Randomly sample two matched pairs of SIFT features from the input data.
  (6) $$\begin{array}{c} \left\{p_{a}=\left(x_{pa},y_{pa}\right),q_{a}=\left(x_{qa},y_{qa}\right)\right\}\\ \{p_{b}=\left(x_{pb},y_{pb}\right),q_{b}=\left(x_{qb},y_{qb}\right)\}. \end{array}$$
  Calculate the four variables in the model $T^{12,\mathrm{guess}}$ with the following equations.
  (7) $$s=\frac{|\sqrt{{\left(x_{qb}-x_{qa}\right)}^{2}+{\left(y_{qb}-y_{qa}\right)}^{2}}|}{|\sqrt{{\left(x_{pb}-x_{pa}\right)}^{2}+{\left(y_{pb}-y_{pa}\right)}^{2}}|}$$
  (8) $$\begin{array}{c} \theta =\arctan\left(\frac{y_{qb}-y_{qa}}{x_{qb}-x_{qa}}\right)-\arctan\left(\frac{y_{qb}-y_{pa}}{x_{pb}-x_{pa}}\right) \end{array}$$
  (9) $$\left[\begin{array}{c} t_{x}\\ t_{y} \end{array}\right]=\left[\begin{array}{c} x_{qa}\\ y_{qa} \end{array}\right]-s\left[\begin{array}{cc} \cos\theta & -\sin\theta \\ \sin\theta & \cos\theta \end{array}\right]\left[\begin{array}{c} x_{pa}\\ y_{pa}. \end{array}\right]$$
Step 2 Test other feature pairs ${\left\{p_{i}=\left(x_{pi},y_{pi}\right),q_{i}=\left(x_{qi},y_{qi}\right)\right\}}_{i=1,2,\cdots ,N}$ and transform the pairs by Equations (5)–(9) as follows.
  (10) $$\begin{array}{cc} v_{qi} & =\left[\begin{array}{c} x_{qi}\\ y_{qi} \end{array}\right]\\ v_{pi}^{\prime } & =s\left[\begin{array}{cc} \cos\theta & -\sin\theta \\ \sin\theta & \cos\theta \end{array}\right]\left[\begin{array}{c} x_{pi}\\ y_{pi} \end{array}\right]+\left[\begin{array}{c} t_{x}\\ t_{y}. \end{array}\right] \end{array}$$
  If the transformed pair satisfies
  (11) $$|v_{qi}-v_{pi}^{\prime }|<\epsilon ,$$
  then the pair conforms to the guess model. ϵ is a parameter for selecting data. The number of pairs that match the guess model is recorded. If that number exceeds the highest number previously recorded, the current model is updated as the ‘best’ model $T^{12,\mathrm{best}}$.
Step 3 Repeat Steps 1 and 2 until the maximum number of iterations is reached and output the best model $T^{12,\mathrm{best}}$ as $T_{\mathrm{RANSAC}}$.

Once we have obtained $T_{\mathrm{RANSAC}}$, the two maps can be overlapped through the transformation. The quality of the transformation is then evaluated using an acceptance index and a threshold $\omega_{\mathrm{thres}}$. If the index exceeds the threshold (in this study, we use 0.95), an entropy filter [17] is applied to adjust the map probabilities and combine the maps into a global map. A higher entropy value corresponds to a higher level of uncertainty. Thus, the entropy filter selects probabilities with low uncertainty [17].

Let us assume that the occupied probabilities of a grid $m_{ij}=M\left(x,y\right)$ in the overlapping areas of two maps $M_{1}$ and $M_{2}$ are $p(M_{1}\left(x,y\right)=1)$ and $p(M_{2}\left(x,y\right)=1)$, respectively. We can calculate the combined probability $p(M_{\mathrm{combined}}\left(x,y\right)=1)$ by updating the occupancy grid mapping in terms of the log probabilities [21]. This can be done using Equation (12). The corresponding entropy is calculated using Equation (13), and the three entropy values for $p(M_{1}\left(x,y\right)=1)$, $p(M_{2}\left(x,y\right)=1)$, and $p(M_{\mathrm{combined}}\left(x,y\right)=1)$ are listed in Equation (14).

(12) $$\begin{array}{cc} p(M_{\mathrm{combined}}\left(x,y\right)=1) & =\frac{Odd(M_{\mathrm{combined}}\left(x,y\right)=1)}{1+Odd(M_{\mathrm{combined}}\left(x,y\right)=1)}\\ \log Odd(M_{\mathrm{combined}}\left(x,y\right)=1) & =\log Odd(M_{1}\left(x,y\right)=1)+\log Odd(M_{2}\left(x,y\right))\\ Odd(M_{1}\left(x,y\right)=1) & =\frac{p(M_{1}\left(x,y\right)=1)}{1-p(M_{1}\left(x,y\right)=1)}\\ Odd(M_{2}\left(x,y\right)=1) & =\frac{p(M_{2}\left(x,y\right)=1)}{1-p(M_{2}\left(x,y\right)=1)} \end{array}$$

(13) $$\begin{array}{c} H(M(x,y)=1)=-p(M(x,y)=1)\log p(M(x,y)=1)-(1-p(M(x,y)=1))(\log\left(1-p(M(x,y)=1)\right)) \end{array}$$

(14) $$\begin{array}{cc} H(M_{1}\left(x,y\right)=1)= & -p\left(M_{1}\left(x,y\right)=1\right)\log p(M_{1}\left(x,y\right)=1)\\ \; & -\left(1-p\left(M_{1}\left(x,y\right)=1\right)\right)\left(\log\left(1-p\left(M_{1}\left(x,y\right)=1\right)\right)\right)\\ H(M_{2}\left(x,y\right)=1)= & -p\left(M_{2}\left(x,y\right)=1\right)\log p(M_{2}\left(x,y\right)=1)\\ & -\left(1-p\left(M_{2}\left(x,y\right)=1\right)\right)\left(\log\left(1-p\left(M_{2}\left(x,y\right)=1\right)\right)\right)\\ H(M_{\mathrm{combined}}\left(x,y\right)=1)= & -p\left(M_{\mathrm{combined}}=1\right)\log p(M_{\mathrm{combined}}=1)\\ & -\left(1-p\left(M_{\mathrm{combined}}=1\right)\right)\left(\log\left(1-p\left(M_{\mathrm{combined}}=1\right)\right)\right). \end{array}$$

In overlapping areas, the occupancy probability of grids from different maps are considered together and a grid with low uncertainty is selected as the output.

### 4.2. Proposed Image Pre-Processing Operation

**Figure 4:**
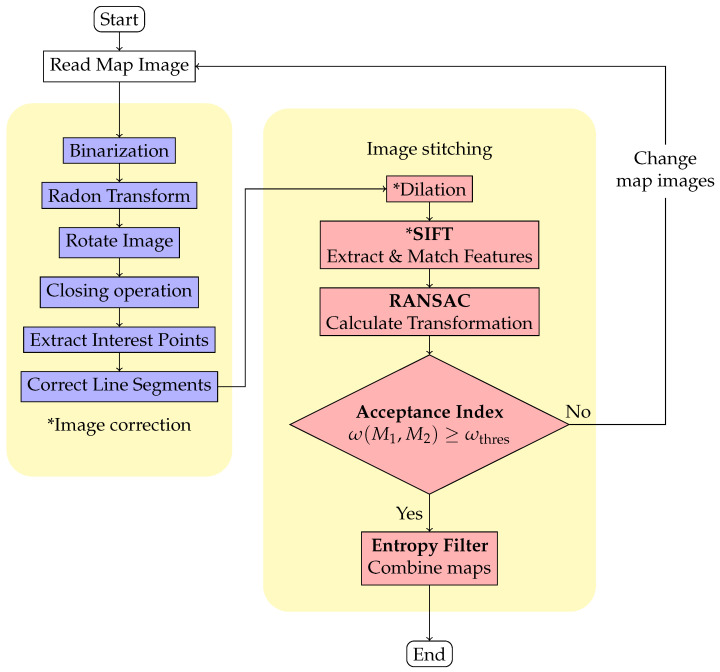
Flowchart of proposed method. Blocks with an asterisk “*” indicate added or slightly modified steps.

Failed map merging can occur due to uncertainty in the map and distortion caused by sensing errors. To tackle this issue, we applied image correction to the maps, which eliminates uncertainty and distortion, and improves the merge result after feature extraction and matching. Our image correction process involves five steps, which are illustrated in the image correction block of Figure 4.

#### 4.2.1. Image Correction Process

To extract features with matching relationships from occupancy grid maps, it is important to avoid using pixel values of unknown regions with high uncertainty. In order to get around this, we use a method called binarization that separates the map’s regions into “occupied” and “non-occupied” sections. The “occupied” section includes the original occupied area, while the “non-occupied” section consists of the original free and unknown areas.

**Figure 5:**
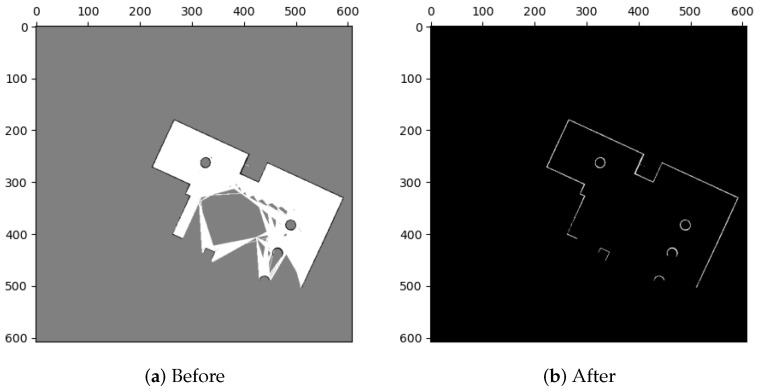
Binarization.

In binarization, each grid is classified using a threshold value $B_{\mathrm{thres}}$. If the pixel value of a grid M(x,y) is less than $B_{\mathrm{thres}}$, it is classified as occupied and assigned a new value of 255. If M(x,y) is greater than or equal to $B_{\mathrm{thres}}$, it is classified as non-occupied, and assigned a new value of 0, as shown in Equation (15). After binarization, the map only contains two types of grids, as illustrated in Figure 5.

(15) $$\begin{array}{c} M\left(x,y\right)=\left\{\begin{array}{cccc} 255\; & \left(\mathrm{Occupied}\right) & \; & \mathrm{if}\;M\left(x,y\right)<B_{\mathrm{thres}}\\ 0\; & \left(\mathrm{Non-occupied}\right) & \; & \mathrm{else}. \end{array}\right. \end{array}$$

**Figure 6:**
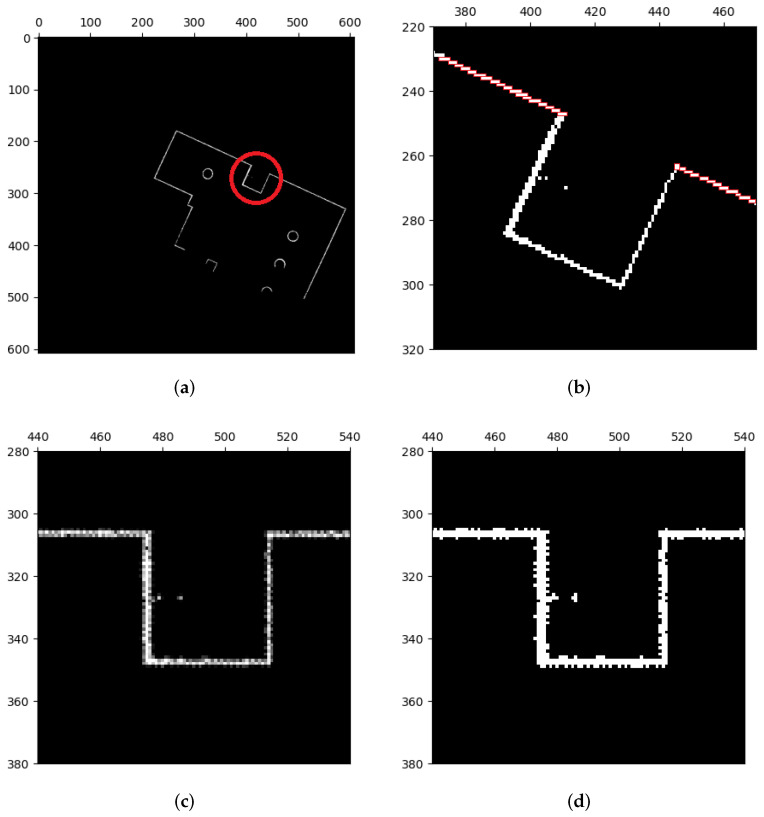
Rotation of map images. (**a**) Binarized map. Stair shapes of linear features are marked with a red circle. (**b**) The zoomed part of (**a**). (**c**) The rotated image of (**b**). Stair shapes are roughly eliminated, but some pixel values of grids are unequal to 0 or 255 due to interpolation. (**d**) Implement binarization to classify pixel values. Stair shapes are still eliminated but some uneven burrs happen instead. These defects will be eliminated by image closing.

Figure 6b illustrates what can happen when barriers like linear walls on a map are not parallel or perpendicular to the coordinate axes: they can take the form of stairs. We rotate the maps to align linear features with the coordinate axes in order to improve feature extraction. The Radon transform, which is applied to the map, is used to determine the rotation’s angle (ref. [22]). By performing line integration on *f*, the Radon transform turns the map image into a two-dimensional linear function Rf, $M\overset{f}{\to }Rf$, as shown in Equation (16).

(16) $$\begin{array}{c} Rf=R\left(s,\alpha \right)=\int \int_{R^{2}}f\left(x,y\right)\delta \left(x\cos\left(\alpha \right)+y\sin\left(\alpha \right)-s\right)dxdy. \end{array}$$

**Figure 7:**
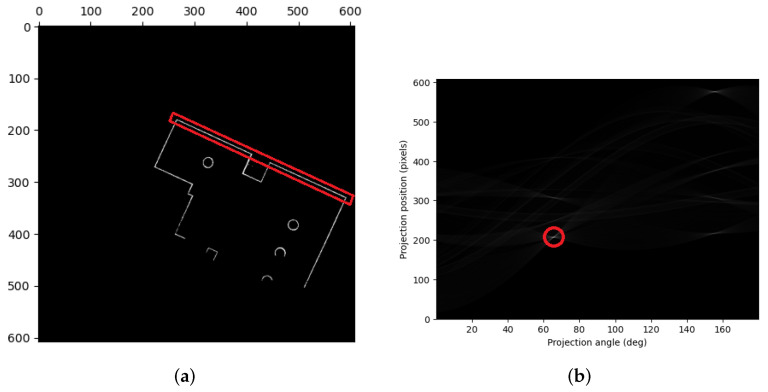
Radon transform. (**a**) Map image. The longest line is marked with a red rectangle. (**b**) Two-dimensional space linear function Rf. The largest value is marked with a red circle.

The longest line in the original map image corresponds to the greatest value of the linear function Rf following the Radon transform on the occupancy grid map, as shown in Figure 7. The rotation angle for the map can be calculated as the angle between this longest line and the coordinate axis. However, as illustrated in Figure 6c, some grids’ pixel values may no longer be equal to 0 or 255 as a result of rotation’s interpolation calculations. Therefore, as shown in Figure 6d, we apply extra binarization to make sure that the map image only comprises occupied and unoccupied areas. We use methods from image morphology [23] to fix instances where the map image has uneven burrs and imperfections as a result of prior operations.

The dilation operation from image morphology moves the structuring element *S* across each grid M(x,y) of the image *M* when given an image *M* and *S*. When *S* intersects with the grids surrounding M(x,y), the value of M(x,y) is set to 255; otherwise, it is set to 0. While the foreground regions of image *M* are expanded by the dilation operation, the erosion operation, reduces the foreground regions of the image *M*. Erosion process sets the value of M(x,y) to 255 only if *S* belongs to grids surrounding M(x,y); otherwise, it is set to 0.

**Figure 8:**
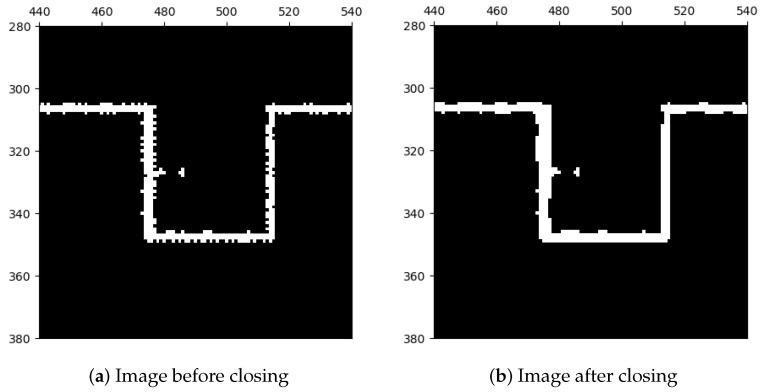
Closing operation.

To improve the image, we performed a closing operation as shown in Figure 8, which applies dilation followed by erosion. This operation expands the foreground regions (i.e., the occupied image areas), fills gaps and defects by dilation, and then restores the area to its original size by erosion. The closing operation can be represented mathematically by Equation (17), where the ⊕, ⊖, and • operators represent dilation, erosion, and closing, respectively.

(17) $$\begin{array}{cc} M⊕S & =\{z|\hat{{\left(S\right)}_{z}}\cap M\neq \phi \}\\ M⊖S & =\{z|\hat{{\left(S\right)}_{z}}\subseteq M\}\\ M•S & =(M⊕S)⊖S. \end{array}$$

**Figure 9:**
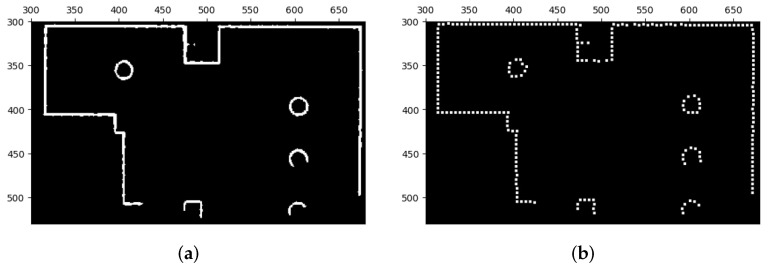
Extraction of interest points. (**a**) Image before the extraction of interest points. (**b**) Image after the extraction of interest points.

**Figure 10:**
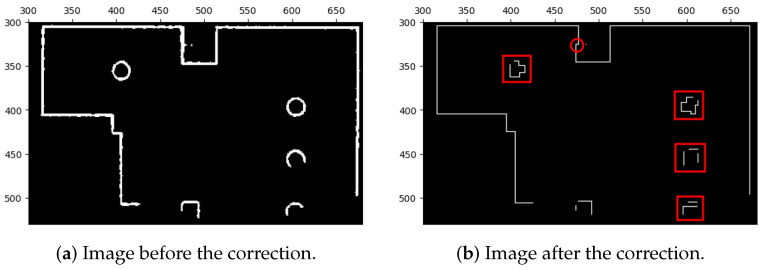
Correction of line segments of map images with extracted interest points. Certain geometric shapes that are sensitive to orientation might be influenced by the correction, as indicated by the red boxes and circles. Nevertheless, we can still extract comparable features and subsequently match them during the image stitching process. This is possible because the majority of extracted features are derived from the corrected line segments, while only a small portion originates from these orientation-sensitive shapes.

After performing the closing operation, the preliminary correction of the lines in the occupied areas has been completed. However, to further improve the quality of the lines and increase their straightness, remove defects, and normalize their widths, we applied another correction method before using the feature extraction algorithm. This correction method consists of two parts—the first part involves sampling interest points from the current lines as depicted in Figure 9 and the second part involves redrawing new lines based on these interest points as shown in Figure 10.

The extraction of interest points from the lines is performed by Algorithm 1, which creates a new image $M^{\prime }$ with all interest points. To achieve this, the structuring element *S* is moved across the original image *M* in a similar way to previous image morphology operations. If *S* intersects with the grids surrounding M(x,y), a marker value of 255 is assigned to the grid $M^{\prime }\left(x,y\right)$, and the original grids surrounding M(x,y) are deleted by setting their values to 0. After the movement of the structuring element *S* is completed on each grid of *M*, all interest points are identified and marked on image $M^{\prime }$.
**Algorithm 1** Extraction of interest points of map images.**Input:*** M*: map image; *S*: structuring element; **Output: **$M^{\prime }$: map image with interest points
1:r, c ← size(*M*)2:$M^{\prime }\leftarrow$ new matrix(r,c)3:**for **x=1 to *r* **do**4:      **for** y=1 to *c* **do**5:            $M_{s}\leftarrow$ surrounding of M(x,y)6:            **if** $S\subseteq M_{s}$ **then**7:                  $M^{\prime }\left(x,y\right)\leftarrow 255$8:                  surrounding of M(x,y)←09:            **end if**10:      **end for**11:**end for**12:**return** $M^{\prime }$

Algorithm 2 is the final step of our proposed image correction process, presented in Figure 4, which aims to correct the line segments in the occupied area. The algorithm takes the image $M^{\prime }$ with interest points and the image *M* after closing as inputs, along with the distance parameter *L*, to ensure that the new line segments resemble the original data with some uncertainty and are parallel to the coordinates. The algorithm finds points on the map that form line segments and corrects them using the following five steps:

Step 1 Find the reference point $M^{\prime }\left(x_{i},y_{i}\right)$ closest to the origin using the Manhattan distance in Equation (18).
  (18) $$\left(x_{i},y_{i}\right)=\arg\min_{x,y}\;|x-x_{o}|+|y-y_{0}|;$$
Step 2 Find the target point $M^{\prime }\left(x_{t},y_{t}\right)$ closest to the reference point as presented in Equation (19).
  (19) $$\left(x_{t},y_{t}\right)=\arg\min_{x,y}\;|x-x_{i}|+|y-y_{i}|;$$
Step 3 Connect $M^{\prime }\left(x_{i},y_{i}\right)$ and $M^{\prime }\left(x_{t},y_{t}\right)$ with a line, and all grids ($M^{\prime }\left(x_{1},y_{1}\right),\cdots ,M^{\prime }\left(x_{n},y_{n}\right)$) that the line intersects with are recorded. The position of these *n* grids is then used to calculate the ratio $R_{it}$ of occupied areas on the original image *M*, as shown in Equation (20).
  (20) $$R_{it}=\frac{\sum_{i=1}^{n}M\left(x_{i},y_{i}\right)}{255\times n}.$$
  The line connecting $M^{\prime }\left(x_{i},y_{i}\right)$ and $M^{\prime }\left(x_{t},y_{t}\right)$ was present in the original image and can be corrected if the calculated ratio $R_{it}$ exceeds the defined threshold $R_{thres}$, $R_{it}>R_{thres}$. If not, the algorithm picks a new target point and repeats Step 2 in that case.
Step 4 Calculate a vector $v_{it}$ as Equation (21) if the line between $M^{\prime }\left(x_{i},y_{i}\right)$ and $M^{\prime }\left(x_{t},y_{t}\right)$ can be corrected.
  (21) $$v_{it}=\left(x_{t}-x_{i},y_{t}-y_{i}\right).$$
  According to $v_{it}$, we then take $M^{\prime }\left(x_{i},y_{i}\right)$ as a reference and translate $M^{\prime }\left(x_{t},y_{t}\right)$. The translation follows the following rule.
  (1) If $|x_{t}-x_{i}\left|\;<\;\right|y_{t}-y_{i}|$, translate grid $M^{\prime }\left(x_{t},y_{t}\right)$ in *x*-direction. For example, moves $M^{\prime }\left(x_{t},y_{t}\right)$ to $M^{\prime }\left(x_{i},y_{t}\right)$;
  (2) If $|x_{t}-x_{i}\left|\;>\;\right|y_{t}-y_{i}|$, translate grid $M^{\prime }\left(x_{t},y_{t}\right)$ in *y*-direction. For example, moves $M^{\prime }\left(x_{t},y_{t}\right)$ to $M^{\prime }\left(x_{t},y_{i}\right)$;
  (3) If $|x_{t}-x_{i}\left|\;=\;\right|y_{t}-y_{i}|$, add an additional grid as a target point according to the previous direction and perform the translation again. For example, if the previous direction is:
    - *x*-direction, then add a new grid $M^{\prime }\left(x_{i},y_{t}\right)$ as a new target point;
    - *y*-direction, then add a new grid $M^{\prime }\left(x_{t},y_{i}\right)$ as a new target point;
    - none (no previous direction), then abandon the translation and select another reference and target points.
  In the aforementioned translation, shifts or additions of grids are performed. To ensure that the correction does not differ excessively from the original image, we again connect the grid $M^{\prime }\left(x_{i},y_{i}\right)$ and the shifted (or added) grid $M^{\prime }\left(x_{i},y_{t}\right)$ (or $M^{\prime }\left(x_{t},y_{i}\right)$) on a line, record all *n* grids that the line passes through, and calculate the ratio $R_{it^{\prime }}$ of occupied areas at the same positions on original image *M*. This calculation is the same as Equation (20). If ratio $R_{it^{\prime }}$ is greater than $R_{thres}$, the correction is accepted and the occupied grids on the connected line will be stored to corrected image $M_{\mathrm{correct}}$. If not, the correction is rejected and the original information from image *M* will be stored to corrected image $M_{\mathrm{correct}}$.
Step 5 According to the result of Step 4, the correction point $M^{\prime }\left(x_{i},y_{t}\right)$ (or $M^{\prime }\left(x_{t},y_{i}\right)$) and can be used as a new reference point to find a new adjacent target point. Steps 3 to 5 are repeated until the correction is completed. Once the correction is completed, the corrected image, $M_{\mathrm{correct}}$, is output as the result.

**Algorithm 2** Corrected occupancy grid maps with interest points.**Input: ***M*: map image; $M^{\prime }$: map image with interest points; *L*: distance parameter; **Output: **$M_{\mathrm{correct}}$: map image after correction 1:r, c ← size(*M*)2:$M_{\mathrm{correct}}\leftarrow$ new matrix(r,c)3:direction ← none4:$p_{start}^{\prime }\leftarrow$ search for the nearest point of interest to the origin on $M^{\prime }$5:**while** interest points exist **do**6:        $p_{target}^{\prime }\leftarrow$ search for the nearest point of interest to $p_{start}^{\prime }$ on $M^{\prime }$ within distance *L*7:        **if** $p_{target}^{\prime }$ exists **then**8:              $p_{start},p_{target}\leftarrow$ find points with the same position of $p_{start}^{\prime }$ and $p_{target}^{\prime }$ on *M*9:              $l_{st}\leftarrow$ make a line from $p_{start}$ to $p_{target}$10:            **if** all points on $l_{st}$ = 255 **then**11:                  $\left(dx,dy\right)\leftarrow \left(p_{target}.x-p_{start}.x,p_{target}.y-p_{start}.y\right)$12:                  **if** |dx|>|dy| **then**13:                        $p_{correct}\leftarrow \left(p_{start}.x,p_{target}.y\right)$14:                        $l_{sc}\leftarrow$ make a line from $p_{start}$ to $p_{correct}$15:                        **if** all points on $l_{sc}$ = 255 **then**16:                              $M_{\mathrm{correct}}\leftarrow$ set the value of all points on $l_{sc}$ to 25517:                              direction ← toward_y18:                              $M^{\prime }\leftarrow$ delete $p_{start}^{\prime }$ and $p_{target}^{\prime }$ on $M^{\prime }$19:                              $p_{start}^{\prime }\leftarrow p_{correct}$20:                        **else**21:                              $M_{\mathrm{correct}}\leftarrow$ set the value of all points on $l_{st}$ to 25522:                              direction ← None23:                        **end if**24:                  **else if** |dx|<|dy| **then**25:                        $p_{correct}\leftarrow \left(p_{target}.x,p_{start}.y\right)$26:                        $l_{sc}\leftarrow$ make a line from $p_{start}$ to $p_{correct}$27:                        **if** all points on $l_{sc}$ = 255 **then**28:                              $M_{\mathrm{correct}}\leftarrow$ set the value of all points on $l_{sc}$ to 25529:                              direction ← toward_x30:                              $M^{\prime }\leftarrow$ delete $p_{start}^{\prime }$ and $p_{target}^{\prime }$ on $M^{\prime }$31:                              $p_{start}^{\prime }\leftarrow p_{correct}$32:                        **else**33:                              $M_{\mathrm{correct}}\leftarrow$ set the value of all points on $l_{st}$ to 25534:                              direction ← None35:                        **end if**36:                  **else**37:                        **if** direction = toward_y **then**38:                              $p_{correct}\leftarrow \left(p_{start}.x,p_{target}.y\right)$39:                              $l_{sc}\leftarrow$ make a line from $p_{start}$ to $p_{correct}$40:                              **if** all points on $l_{sc}$ = 255 **then**41:                                    $M_{\mathrm{correct}}\leftarrow$ set the value of all points on $l_{sc}$ to 25542:                                    direction ← toward_y43:                                    $M^{\prime }\leftarrow$ delete $p_{start}^{\prime }$ on $M^{\prime }$44:                                    $p_{start}^{\prime }\leftarrow p_{correct}$45:                              **else**46:                                    $M_{\mathrm{correct}}\leftarrow$ set the value of all points on $l_{st}$ to 25547:                                    direction ← None48:                              **end if**49:                        **else if** direction = toward_x **then**50:                              $p_{correct}\leftarrow \left(p_{target}.x,p_{start}.y\right)$51:                              $l_{sc}\leftarrow$ make a line from $p_{start}$ to $p_{correct}$52:                              **if** all points on $l_{sc}$ = 255 **then**53:                                    $M_{\mathrm{correct}}\leftarrow$ set the value of all points on $l_{sc}$ to 25554:                                    direction ← toward_x55:                                    $M^{\prime }\leftarrow$ delete $p_{start}^{\prime }$ on $M^{\prime }$56:                                    $p_{start}^{\prime }\leftarrow p_{correct}$57:                              **else**58:                                    $M_{\mathrm{correct}}\leftarrow$ set the value of all points on $l_{st}$ to 25559:                                    direction ← None60:                              **end if**61:                        **else**62:                              $M^{\prime }\leftarrow$ delete $p_{start}^{\prime }$ on $M^{\prime }$63:                              $p_{start}^{\prime }\leftarrow$ for the nearest point of interest to the origin on $A^{\prime }$64:                        **end if**65:                  **end if**66:            **end if**67:      **else**68:            $M^{\prime }\leftarrow$ delete $p_{start}^{\prime }$ on $M^{\prime }$69:            $p_{start}^{\prime }\leftarrow$ for the nearest point of interest to the origin on $M^{\prime }$70:      **end if**71:**end while**72:**return** $M_{\mathrm{correct}}$

#### 4.2.2. Image Stitching with ICP

After the image correction, the map images only contain line segments with consistent width that are parallel to the coordinate axes. Features from these maps can then be extracted to enable image stitching. However, feature extraction involves the extraction of the extreme points in the scale space of the image, a certain occupancy proportion is required to effectively detect the extreme points in the scale space. In our experiments, an additional dilation operation might be required before extracting features when we have insufficient proportion of occupied areas in the map. In addition, most extracted features are roughly located at the corner or the end of the line segments where their neighborhoods are overly similar, causing the feature descriptors to be similar and preventing matching using the Euclidean distance. Instead, we used brute-force matching, as in Equation (22).

(22) $$\{\left\{p_{1},q_{1}\right\},\ldots ,\left\{p_{1},q_{m}\right\},\ldots ,\left\{p_{n},q_{1}\right\},\ldots ,\left\{p_{n},q_{m}\right\}\},$$

where ${\left\{p_{i}\right\}}_{i=1,\ldots ,n}$ and ${\left\{q_{j}\right\}}_{j=1,\ldots ,m}$ are features from maps $M_{1}$ and $M_{2}$, respectively.

When using RANSAC to merge corrected map images, slight deviations may occur. Therefore, we first performed an initial alignment of the maps using the RANSAC model $T_{\mathrm{RANSAC}}$ and then applied the Iterative Closest Point (ICP) algorithm to achieve a more precise alignment.

ICP is a point cloud matching and alignment method that utilizes the least square difference to calculate a spatial transformation and minimize the distance between two input point clouds to achieve alignment [24,25]. In our approach, we use the occupied grids ($P_{\mathrm{occ}}=p_{\mathrm{occ},1},\ldots ,p_{\mathrm{occ},n}$, $Q_{\mathrm{occ}}=q_{\mathrm{occ},1},\ldots ,q_{\mathrm{occ},m}$) from the original maps ($M_{1}$, $M_{2}$) as point clouds and applied the ICP algorithm to obtain a model for the second alignment. To ensure good initial conditions, we transformed the point clouds, $P_{\mathrm{occ}}$, as described in Equation (23).

(23) $$P_{\mathrm{occ}}^{\prime }=T_{\mathrm{RANSAC}}\times P_{\mathrm{occ}}=\left\{p_{\mathrm{occ},1}^{\prime },\ldots ,p_{\mathrm{occ},n}^{\prime }\right\}.$$

During each iteration of the ICP algorithm, every point in the set $P_{\mathrm{occ}}^{\prime }$ is matched with its corresponding point in the point set $Q_{\mathrm{occ}}$ to establish a correspondence relationship of ${p_{\mathrm{occ},i}^{\prime },q_{\mathrm{occ},i}}_{i=1,\ldots ,N_{p}}$. Using this correspondence relationship, the ICP model for the second alignment can be obtained by solving Equation (24).

(24) $$\begin{array}{cc} R^{*},t^{*} & =\arg\min_{R,t}\sum_{i=1}^{N_{p}}\left|\right|q_{\mathrm{occ},i}-\left(Rp_{\mathrm{occ},i}^{\prime }+t\right){\left|\right|}^{2}\\ T_{\mathrm{ICP}} & =\left[\begin{array}{c} \begin{array}{cc} R^{*} & t^{*}\\ 0 & 1\\ . \end{array} \end{array}\right] \end{array}$$

$R^{*}$ and $t^{*}$ are rotation and translation matrices, respectively. With $T_{\mathrm{RANSAC}}$ and $T_{\mathrm{ICP}}$, a final model is obtained by combining these two models:

(25) $$T^{12}\left(s,\theta ,t_{x},t_{y}\right)=T_{\mathrm{ICP}}\times T_{\mathrm{RANSAC}}.$$

## 5. Results & Discussion

In this section, we provide a comprehensive performance comparison between the utilization of our proposed image pre-processing method and the absence of such pre-processing in two distinct cases, as detailed and analyzed in Section 5.1 and Section 5.2, respectively. Additionally, we present the corresponding merging results for each case to support our findings. Additionally, both of these cases are carried out within the Robotic Operating System (ROS) [26] and the Gazebo virtual world [27]. Moreover, we utilize various open-source algorithms, including OpenCV [28] for feature extraction and Gmapping-based SLAM for mapping the environment.

### 5.1. Scenario 1: Performance of Our Proposed Method

**Figure 11:**
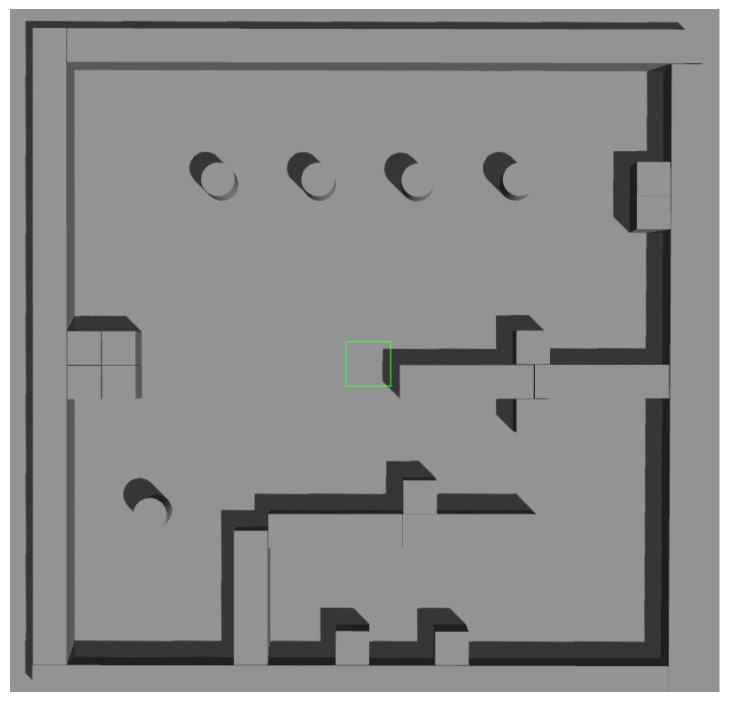
Simulation environment of Scenario 1.

**Figure 12:**
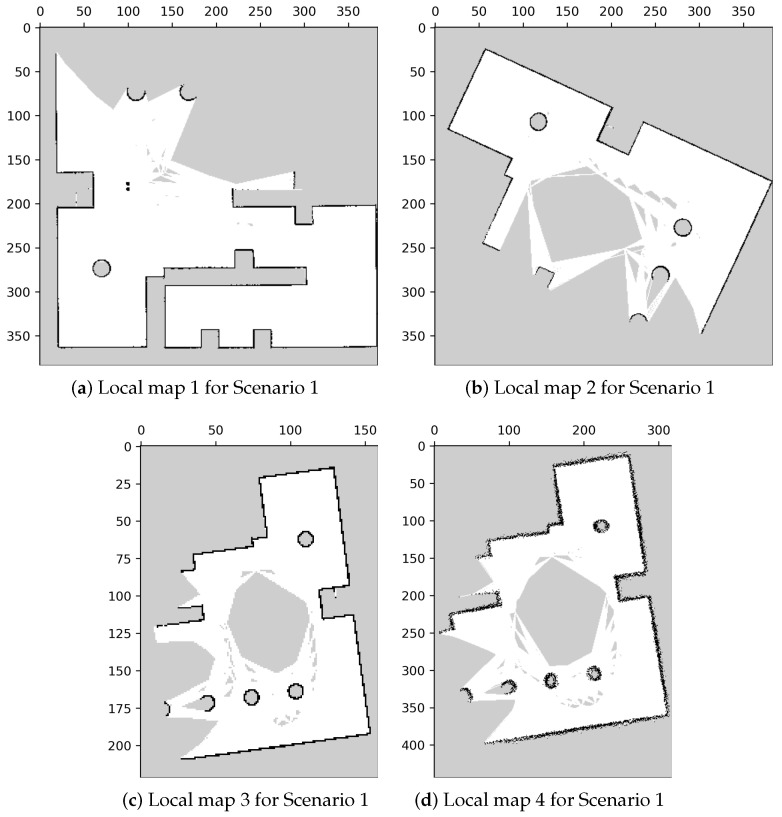
Local maps of the environment in Scenario 1.

A simulation environment was created as shown in Figure 11. In this environment, we created four local maps with different parameters, as illustrated in Figure 12, and the specific parameters are listed in Table 1.

**Figure 13:**
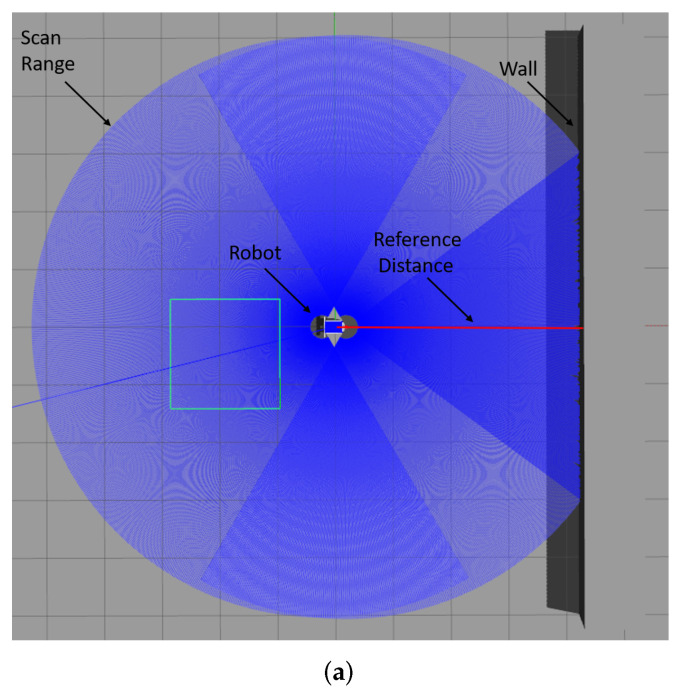

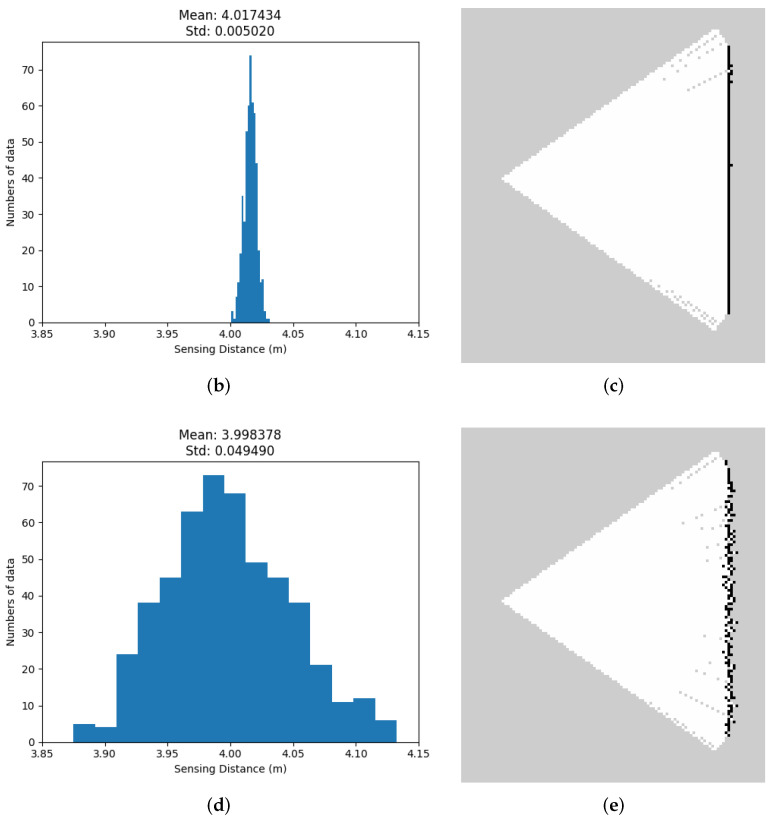
The impact of sensing errors on mapping accuracy. As the standard deviation of LiDAR measurements increases, the resulting occupancy grid maps become more distorted and less accurate. (**a**) Simulation environment for lidar sensing errors. (**b**) Standard deviation of measurement of LiDAR = 0.005. (**c**) Standard deviation = 0.005. The line of occupied grids is straight. (**d**) Standard deviation of measurement of lidar = 0.05. (**e**) Standard deviation = 0.05. The line of occupied grids is distorted.

In Figure 12, the displayed maps exhibit variations in coordinates due to differences in rotation and translation resulting from mapping from various origin positions. Additionally, we manipulated the resolutions of the maps to create variations in their scales. Moreover, we investigated the impact of sensing errors on the map quality, as depicted in Figure 13. In Figure 13a, the robot is positioned at the center, and the blue area represents the LiDAR scanning range. The long object on the right depicts the wall being measured, with the red line indicating the reference distance of 4 m from the robot to the wall. We adjusted the LiDAR signal with different sensor errors, represented by standard deviation, and mapped the wall into occupancy grid maps. The effects of sensing errors can be observed in Figure 13b–e. Mapping with a lower standard deviation of LiDAR measurement (Figure 13) resulted in a more accurate representation of the wall (Figure 13c). Conversely, mapping with a higher standard deviation of LiDAR measurement (Figure 13d) led to a distorted representation of the wall (Figure 13e). These findings highlight the importance of low standard deviation in LiDAR measurements for constructing accurate maps.

Table 2 outlines the relationships between the local maps to be merged, encompassing three distinct data sets. The first set involves merging maps with differing coordinates, while the second set introduces an additional scale difference. The third set focuses on the impact of sensing errors. In the subsequent sections, we will discuss the merging outcomes with and without the utilization of our proposed method.

#### 5.1.1. Results without Image Pre-Process—Mills’ Method [16]

**Figure 14:**
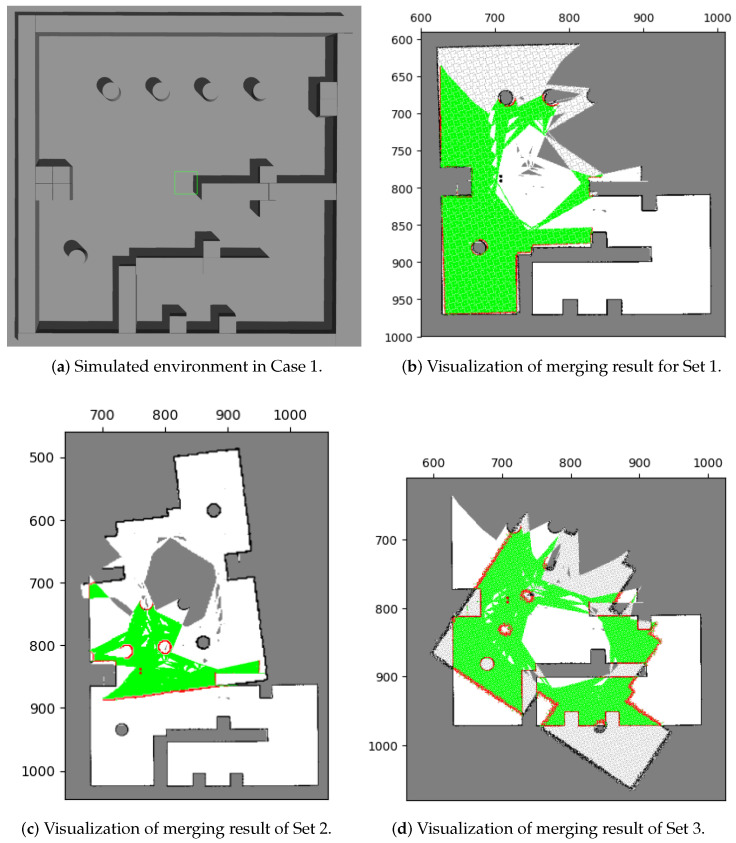
Merging results with the existing method in test case 1. In (**b**–**d**), the green areas represent correct pairings of grids, whereas the red areas represent incorrect pairings. Among these results, only the result of Set 1 correspond to the environment (**a**).

The merging results for the three data sets without the image pre-process (i.e., using Figure 3) are shown in Figure 14, and the corresponding acceptance indices are listed in Table 3.

Only data set 1 (Figure 14a) produces a matching result with the environment. To evaluate the merging performance, Table 3 shows the acceptance indices for the three data sets. The results suggest that a successful merging threshold should fall between 0.966 and 0.935. That is the reason we set the merging threshold, $\omega_{\mathrm{thres}}$, to 0.95 throughout the study.

**Figure 15:**
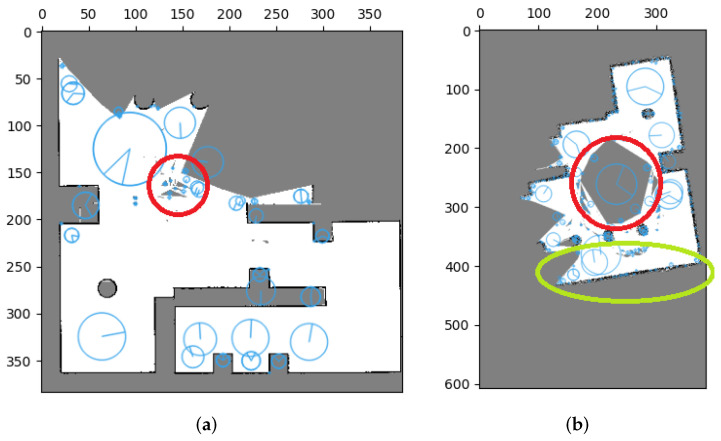
Error effect for SIFT features in occupancy grid maps. Uncertainty can lead to the presence of redundant features (indicated by red circles), while distortion in occupied areas can also result in the same issue (highlighted by green circles). (**a**) Error effect for SIFT features in Local map 1. (**b**) Error effect for SIFT features in Local map 4.

Mills’ method showed ineffective results when merging these occupancy grid maps, mainly due to feature matching errors. During feature extraction, extreme points are calculated based on the value and distribution of pixels, leading to the inclusion of unknown areas with high uncertainty and generating unnecessary features without matching relationships between maps. This issue is highlighted in Figure 15 where red circles indicate these errors. Furthermore, sensing errors can also result in the extraction of features without matching relationships. A smaller number of features were extracted in the occupied area of Figure 15a, while a larger number of features were extracted in the occupied area of Figure 15b (marked in green circles). These feature extraction errors lead to ineffective map merging, as evidenced by our results.

Consequently, inaccurate matching relations can lead to errors in calculating the relative transformation and ultimately result in unsuccessful merges. However, our proposed method provides an effective solution to address this issue. We performed tests on the same datasets, applying our image pre-processing technique before utilizing Mills’ method. The results of these tests will be thoroughly examined and discussed in Section 5.1.2.

#### 5.1.2. Results with Image Pre-Process

**Figure 16:**
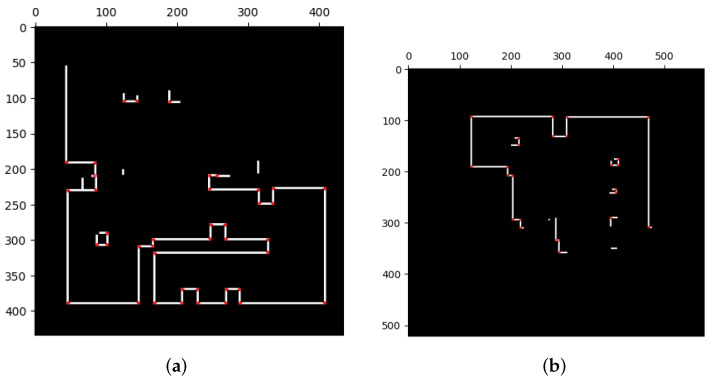
Evaluation of SIFT feature extraction performance with image pre-processing in occupancy grid maps. The extracted features are strategically located at the corners of occupied areas, effectively mitigating the influence of uncertainty and sensing errors. (**a**) Extraction of SIFT features in corrected Local map 1. (**b**) Extraction of SIFT features in corrected Local map 3.

**Figure 17:**
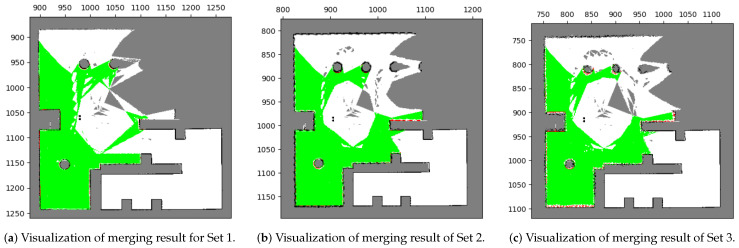
Merging results with our proposed method in test case 1. All the results of the three sets correspond to the environment [Figure 14a].

Figure 16 presents the results of feature extraction after applying our proposed image pre-processing. It is notable that features are selectively extracted only at the corners of occupied areas. Unlike Figure 15, this approach effectively excludes the influence of uncertainties and sensing errors on feature extraction, making it more efficient for true matches of features and the subsequent stitching method. Furthermore, Figure 17 displays the merging results for the three datasets obtained by applying our image pre-processing operation before utilizing Mills’ method (as depicted in Figure 4). The corresponding acceptance indices are presented in Table 4. The results demonstrate a close resemblance to the characteristics of the simulated environment. Moreover, acceptance indices surpassing the predetermined threshold ($\omega_{\mathrm{thres}}=0.95$) signify successful merges. These findings validate that our proposed operation effectively eliminates the impact of sensing errors and uncertainties in occupancy grid maps, resulting in accurate feature matching and correct map merging. Furthermore, Table 5 illustrates the superior performance of the stitching method when coupled with our image pre-processing operation compared to the method without such an operation.

Additionally, to demonstrate that our proposed method can merge multiple maps simultaneously, we created tests in various environments, which will be discussed in greater detail in the subsequent section.

### 5.2. Scenario 2: Merging Multiple Maps

**Figure 18:**
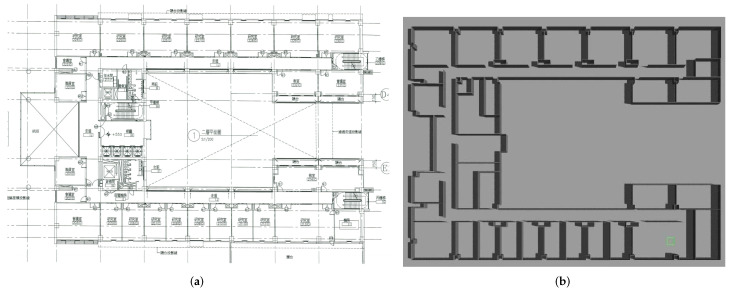
Environment of test case 2. (**a**) Floor plan of Yonglin biomedical engineering hall. (**b**) Simulation environment of Yonglin biomedical engineering hall.

**Figure 19:**
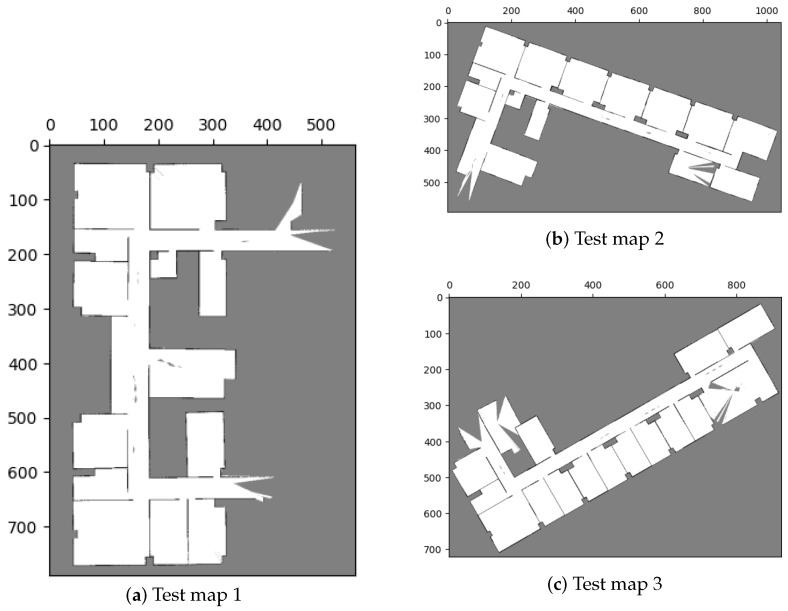
Local maps of Yonglin biomedical engineering hall.

To assess the capability of our method to merge multiple maps simultaneously, we conducted a test in our research office situated in Yonglin Biomedical Engineering Hall, Taipei, Taiwan. In order to expedite the experiment, we simulated the environment using floor plans that faithfully replicated the real-world setting, as shown in Figure 18. Within this simulated environment, we created three distinct local maps, as depicted in Figure 19, and the corresponding parameters can be found in Table 6 and Table 7.

#### Results with the Proposed Method

**Figure 20:**
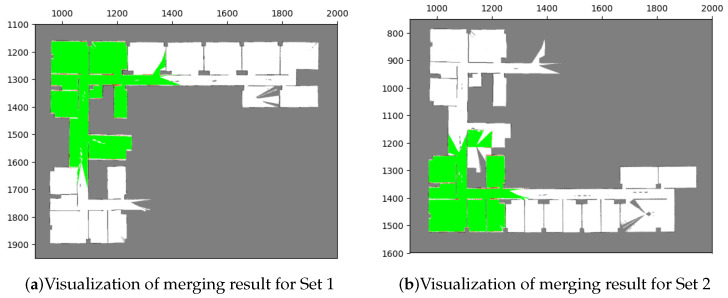
Merging results with our proposed method in test case 2. The visualization indicates that the results conform with the simulation environment (Figure 18).

The merging results for these two sets are illustrated in Figure 20, and it can be observed that most of the overlapping grids have accurate pairings (green areas), with only few grids having incorrect ones (red areas). These red grids are caused by minor errors due to image rotation. The results indicate that the merged maps are consistent with the upper and lower parts of the environment. Additionally, the corresponding acceptance indices are listed in Table 8, both of which exceed the threshold value ($\omega_{\mathrm{thres}}=0.95$), indicating successful merges.

**Figure 21:**
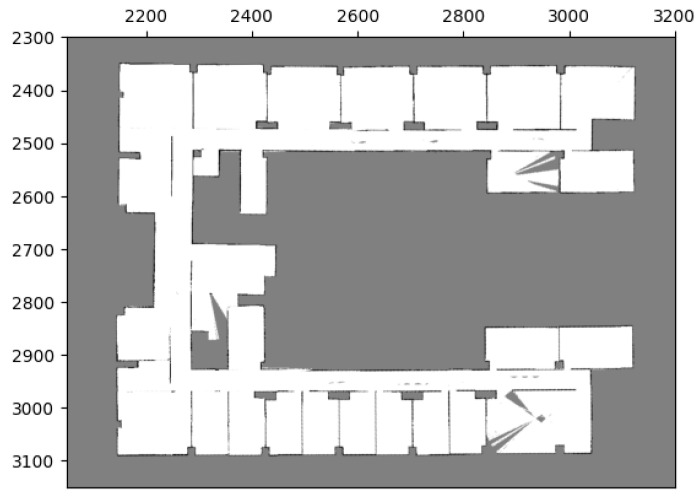
Global maps of Yonglin biomedical engineering hall by merging three local maps in Figure 19.

Finally, the merging operations generate transformations that allow for the combination of the three local maps. By transforming these maps into the same coordinate system and processing their occupancy probabilities through the entropy filter, a global map can be obtained. The resulting global map is presented in Figure 21.

These two cases demonstrate the effectiveness of our proposed method in solving the problem of merging occupancy grid maps.

## 6. Conclusions

In conclusion, we have introduced a novel method for merging occupancy grid maps by utilizing image features to calculate the relative relationships between the maps. Our approach combines the proposed image pre-processing operation with an existing image stitching method, allowing for seamless integration with multi-robot systems without the need for physical interactions, thus enhancing flexibility.

Furthermore, we have identified that occupancy grid maps are susceptible to sensing errors and possess low pixel values, which can result in a reduced number of extracted features or inaccurate feature matching. To address this, we have implemented a pre-processing operation to rectify the map images prior to feature extraction and matching. This operation effectively eliminates the impact of uncertainty and sensing errors, enabling the extraction of features at specific positions. Consequently, this facilitates the accurate calculation of the relative transformation and ensures successful map merging.

We conducted rigorous testing of our method in two distinct environments that resemble typical living spaces. The results of these tests demonstrate the effectiveness of our method in merging maps with varying coordinates, scales, and the ability to merge multiple local maps into a global map simultaneously. Overall, our proposed method offers a robust and flexible approach to map merging for multi-robot systems, providing valuable advancements in this field.

## Figures and Tables

**Table 1:** Parameters used in mapping.

Local Maps	Resolution (Number of Grids/Meter)	Lidar Sensing Error (Standard Deviation, Meter)
Map 1 (Figure 12a)	20	0.005
Map 2 (Figure 12b)	20	0.005
Map 3 (Figure 12c)	10	0.005
Map 4 (Figure 12d)	20	0.05

**Table 2:** Sets of the local maps to be merged.

	Input Map 1	Input Map 2	Relation of Variables
Set 1	Local map 1 (Figure 12a)	Local map 2 (Figure 12b)	Rotation Translation
Set 2	Local map 1 (Figure 12a)	Local map 3 (Figure 12c)	Rotation Translation Scale
Set 3	Local map 1 (Figure 12a)	Local map 4 (Figure 12d)	Rotation Translation Sensing Error

**Table 3:** Corresponding acceptance indices for merged sets in test case 1 by using the existing method.

	Set 1	Set 2	Set 3
Acceptance Index	$\omega_{1}=0.966292$	$\omega_{2}=0.935030$	$\omega_{3}=0.903026$
Human judgment	Success	Failure	Failure

**Table 4:** Corresponding acceptance indices for merged sets in test case 1 by using the reference method.

	Set 1	Set 2	Set 3
Acceptance Index	$\omega_{1}^{\prime }=0.992072$	$\omega_{2}^{\prime }=0.989327$	$\omega_{3}^{\prime }=0.979052$
Human judgment	Success	Success	Success

**Table 5:** Comparison between existing method and our method.

	Existing Method	Our Method	Increased Performance
Set 1	$\omega_{1}=0.966292$	$\omega_{1}^{\prime }=0.992072$	$\frac{\omega_{1}^{\prime }-\omega_{1}}{\omega_{1}}=+2.67\%$
Set 2	$\omega_{2}=0.935030$	$\omega_{2}^{\prime }=0.989327$	$\frac{\omega_{2}^{\prime }-\omega_{2}}{\omega_{2}}=+5.81\%$
Set 3	$\omega_{3}=0.903026$	$\omega_{3}^{\prime }=0.979052$	$\frac{\omega_{3}^{\prime }-\omega_{3}}{\omega_{3}}=+8.42\%$

**Table 6:** Parameters for local maps in test case 2.

Local Maps	Resolution (Number of Grids/Meter)	Lidar Sensing Error (Standard Deviation, Meter)
Map 1 (Figure 19a)	20	0.005
Map 2 (Figure 19b)	20	0.005
Map 3 (Figure 19c)	20	0.005

**Table 7:** Sets of the local maps to be merged.

	Input Map 1	Input Map 2	Relation of Variables
Set 1	Local map 1 (Figure 19a)	Local map 2 (Figure 19b)	Rotation Translation
Set 2	Local map 1 (Figure 19a)	Local map 3 (Figure 19c)	Rotation Translation

**Table 8:** Corresponding acceptance indices for merged sets in test case 2 by using our proposed method.

	Set 1	Set 2
Acceptance Index	$\omega_{1}=0.984169$	$\omega_{2}=0.984952$
Human judgment	Success	Success

## Data Availability

Data sharing not applicable. No new data were created or analyzed in this study.
